# WORK EXPECTATION AND PSYCHOLOGICAL STRESS DURING THE GREAT RECESSION: THE ROLE OF LATE-LIFE EMPLOYMENT TRANSITIONS

**DOI:** 10.1093/geroni/igae098.1663

**Published:** 2024-12-31

**Authors:** Linh Dang, Toni Antonucci, Richard Gonzalez, Philippa Clarke, Carlos Mendes de Leon, Briana Mezuk

**Affiliations:** University of Michigan, Ann Arbor, Michigan, United States; University of Michigan, Ann Arbor, Michigan, United States; University of Michigan, Ann Arbor, Michigan, United States; University of Michigan, Ann Arbor, Michigan, United States; Georgetown University, Washington, District of Columbia, United States; University of Michigan, Ann Arbor, Michigan, United States

## Abstract

Perceived uncertainty about future employment during economic crises negatively affects the mental health of older adults. Moreover, such health impact varies across countries and employment contexts. This study examined the longitudinal association between work expectation and psychological stress among Baby Boomers in the US and South Korea and how employment transitions moderated this association during the Great Recession (2008-2009) and its recovery (2010-2018). Data came from 2006-2018 Health and Retirement Study (n=2,647) and Korean Longitudinal Study of Aging (n=1,454). Perceived expectation of working in the next five years was reported on a probability scale (0-100%). Psychological stress was assessed by the Center for Epidemiologic Studies Depression Scale. Six distinct employment transitions were constructed based on labor force status: entrance to full-time employment, precarious employment, stable full-time employment, self-employment, exit labor force, and being out of labor force. Multivariate mixed-effects logistic regressions were fit, adjusting for sociodemographic and health characteristics. Effect modification by employment transitions was examined via stratification. Higher work expectations were associated with lower odds of psychological stress during recession (OR=0.93 [0.89, 0.98]) and recovery (OR=0.95 [0.92, 0.98]) in US respondents; this association was more pronounced during the recovery in Korea (OR=0.83 [0.79, 0.87]). Among those who transitioned to self-employment, higher expectations were associated with higher odds of stress in US (OR=1.22 [1.02, 1.46]) but lower odds in Korea (OR=0.90 [0.77, 1.05]). Findings suggest opportunities for country-specific workplace policies to support mental health of older adults transitioning to different employment paths, particularly during the uncertain period of economic crisis.

